# Common antibiotics, azithromycin and amoxicillin, affect gut metagenomics within a household

**DOI:** 10.1186/s12866-023-02949-z

**Published:** 2023-08-02

**Authors:** Jessica Chopyk, Ana Georgina Cobián Güemes, Claudia Ramirez-Sanchez, Hedieh Attai, Melissa Ly, Marcus B. Jones, Roland Liu, Chenyu Liu, Kun Yang, Xin M. Tu, Shira R. Abeles, Karen Nelson, David T. Pride

**Affiliations:** 1 Department of Pathology, University of California San Diego, 9500 Gilman Drive, MC 0612, La Jolla, San Diego, CA 92093-0612 USA; 2 Department of Medicine, University of California San Diego, San Diego, CA 92093 USA; 3 Genomic Medicine, J. Craig Venter Institute, La Jolla, CA 92037 USA; 4 Division of Biostatistics and Bioinformatics, Herbert Wertheim School of Public Health and Human Longevity Science, University of California San Diego, San Diego, CA 92093 USA

**Keywords:** Fecal, Gut, Microbiome, Microbiota, Metagenome, Antibiotic perturbations, Antibiotic courses, Resistome, Virus, Virome, Bacteriophage, Macrolide, Beta Lactam

## Abstract

**Background:**

The microbiome of the human gut serves a role in a number of physiological processes, but can be altered through effects of age, diet, and disturbances such as antibiotics. Several studies have demonstrated that commonly used antibiotics can have sustained impacts on the diversity and the composition of the gut microbiome. The impact of the two most overused antibiotics, azithromycin, and amoxicillin, in the human microbiome has not been thoroughly described. In this study, we recruited a group of individuals and unrelated controls to decipher the effects of the commonly used antibiotics amoxicillin and azithromycin on their gut microbiomes.

**Results:**

We characterized the gut microbiomes by metagenomic sequencing followed by characterization of the resulting microbial communities. We found that there were clear and sustained effects of the antibiotics on the gut microbial community with significant alterations in the representations of *Bifidobacterium* species in response to azithromycin (macrolide antibiotic). These results were supported by significant increases identified in putative antibiotic resistance genes associated with macrolide resistance. Importantly, we did not identify these trends in the unrelated control individuals. There were no significant changes observed in other members of the microbial community.

**Conclusions:**

As we continue to focus on the role that the gut microbiome plays and how disturbances induced by antibiotics might affect our overall health, elucidating members of the community most affected by their use is of critical importance to understanding the impacts of common antibiotics on those who take them.

Clinical Trial Registration Number NCT05169255. This trial was retrospectively registered on 23–12-2021.

**Supplementary Information:**

The online version contains supplementary material available at 10.1186/s12866-023-02949-z.

## Background

The human microbiome is a combination of microorganisms (viruses, bacteria, fungi, etc.) living in the human body that together outnumber our own number of cells [1]. There have been varied studies describing the difference in the colonic microbiome according to sex, ethnicity, geographic location [2], as well as co-inhabitants of a household [1]. The microbiome in turn can be affected by underlying comorbidities such as diabetes or inflammatory bowel disease [3], but also by diet [4]. The microbiomes of individuals with more diverse microbial communities are more stable and therefore more resistant to invasion [5], thus a depletion in microbial diversity can negatively impact gut health [6]. In the era of rising antimicrobial resistance, the effect that antibiotics have on human microbiota has been of increasing interest. According to the Centers for Disease Control and Prevention (CDC), as of 2014, more than 260 million courses of antibiotics were prescribed, with at least 30% of the outpatient antibiotic prescriptions analyzed in that year deemed to be unnecessary [7]. And according to the 2019 CDC’s Antibiotic Resistance Threats report, more than 2.8 million antibiotic-resistant infections occur in the US yearly, with a mortality rate of 35,000 people per year [8].

Since the discovery that co-inhabiting individuals tend to share similar microbiomes, there is increasing interest in how social relationships affect the microbiome. We know that close social relationships, especially close spousal relationships, are linked to similar gut microbiomes [9] and skin microbiomes [10]. The benefits of social interactions have become evident as individuals who were cohabiting with their spouse had higher alpha-diversity than individuals who were unmarried and living alone [9]. The link between couples’ microbiomes is so pronounced that one can predict which individuals are in a romantic relationship based on their skin microbiota; for instance, our daily shedding of biological particles has resulted in similar foot microbiome profiles of partners [10]. Microbial communities are even shared amongst dogs and their coinhabiting owners—Song et al. showed that adults with dogs have more diverse skin microbiota that is shared with their dogs [11]. Indeed, it is evident that our ‘microbial cloud’—or the distinct, personalized airborne bacterial emissions that humans release—can play a significant role in bacterial transmission amongst individuals [12].

Bacteria are not the only microorganisms that can be transmitted within a household. In fact, it is estimated that there are over 10^12^ viral particles in the human gut [13], and while they play an important and complex role [14], their transmission mechanism is largely unknown. Several phages have been found capable of transducing antibiotic resistant genes [15]. Recent evidence has even shown that antibiotic resistant bacteria harboring genes that allow immune evasion can be transmitted between humans and household livestock, as seen with methicillin resistant *S. aureus* prophage [16]. Given the transmission of phages amongst the household [17], the interconnectedness of microbial communities between coinhabiting individuals is hard to deny, and the implications of this interconnectedness, especially in regard to antibiotic use, has yet to be fully understood.

The effect of broad-spectrum antibiotics on the human microbiome includes changes in the microbial composition, an increase in antibiotic resistance genes, and an increase in virulence genes [18]; in particular, the spread of AMR genes among pathogens in clinical settings is especially concerning [19]. Current research has mostly focused on antibiotics such as ciprofloxacin and clindamycin [20], which are known to have side effects such as *Clostridium difficile* colitis and lead to slower recovery of the microbiome than other antibiotics [21]. However, as discussed in Abeles et al., the effect of the two most overused antibiotics, azithromycin and amoxicillin, in the human microbiome has not been thoroughly described. Though azithromycin and amoxicillin are widely prescribed [22] for having a milder effect on the gut microbiome, they still significantly decrease the diversity of the gut [6, 23].

This study evaluated 56 subjects, of which 24 households had cohabitants, whereas 8 lived alone. In the cohabitant households consisting of 2 people, one of them took either amoxicillin or azithromycin, while the other took placebo (Vitamin C). The control group of 8 lone individuals did not take either antibiotic or placebo. The same cohort was previously evaluated via 16S profiling (Abeles et al., 2016). Our goals in this study were to show the difference in microbiota of household contacts who simultaneously received antibiotics (amoxicillin vs azithromycin) vs placebo by using metagenomics to understand changes in bacteria abundances at the species level, alterations in gene functions that encode antibiotic resistance, and compositional differences in the phageome that might result from antibiotic perturbations.

## Results

### Subject demographics

We recruited 56 subjects comprising 24 separate households over a 6-month period from the University of California San Diego undergraduate campus (Table S1, Figure S1). There were two separate individuals enrolled from each household, with 1 individual receiving an antibiotic (amoxicillin or azithromycin) and the other receiving a placebo (vitamin C). We included an additional 8 subjects who were not enrolled with a housemate. Antibiotic/placebo were given once per day with half the participants receiving therapy for 3 days and the other half for 7 days. Fecal samples were collected on day 0, day 3, day 7, week 8, and at 6 months.

### Fecal metagenome sequencing

In total 282 samples were sequenced on the Illumina HiSeq. After quality filtering and merging there was a total of 5,568,491,056 reads, with an average of 19,746,422 (± 3,848,408 Standard Deviation, SD) per sample and a median of 20,060,036 reads per sample. The average GC content for all the high-quality reads was 46% (+ / − 1.5% SD) overall. There was no significant difference in GC content among the amoxicillin treated participants, the azithromycin treated participants, or the non-household controls at each time point sampled (data not shown).

### Bacterial relative abundances in response to antibiotics

**Fig. 1:**
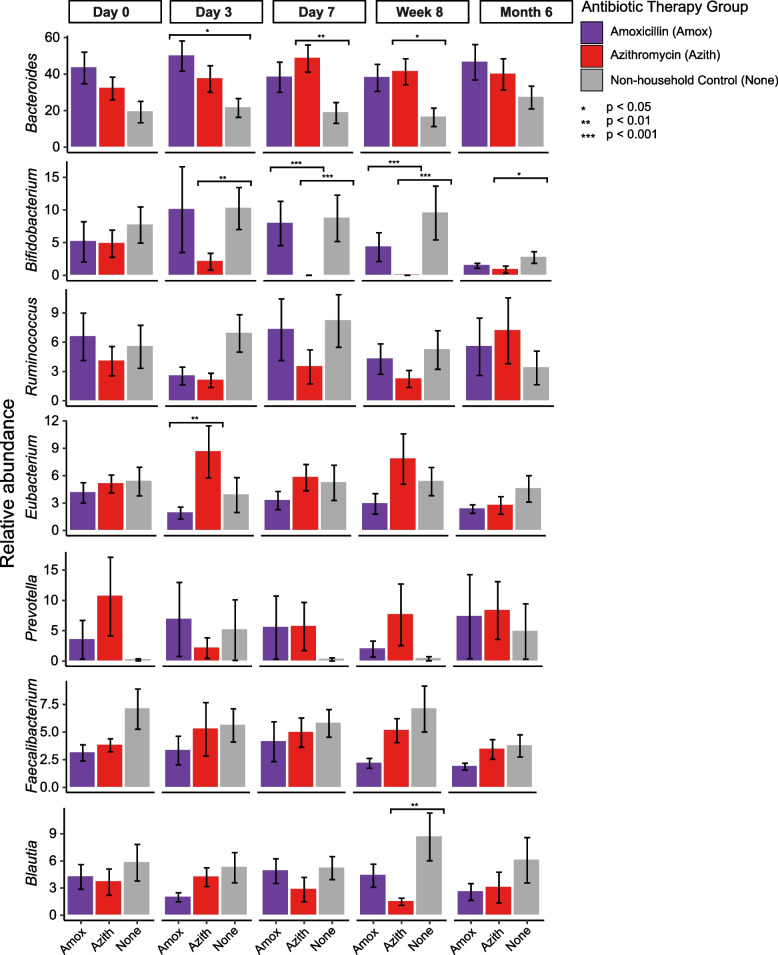
Relative abundance (± standard error) of the most dominant bacterial genera among all amoxicillin treated participants, all azithromycin treated participants, and all non-household controls at each time point sampled. The y-axis represents the relative abundace of the dominant bacterial genera, and the x-axis represents the therapy they received and grouped by the time point sampled. Bars are colored by their antibiotic therapy group (amoxicillin, purple; azithromycin, red; non-household controls, gray). *denotes significance based on Kruskal–Wallis tests with correction via the Holm method

The bacterial genera with the highest relative abundance among all subjects included *Bacteroides* (34.3 ± 25.7% S.D.), *Bifidobacterium* (5.2 ± 10.2% S.D.), *Ruminococcus* (5.0 ± 7.0% S.D.), *Eubacterium* (4.7 ± 5.6% S.D.), *Prevotella* (4.6 ± 13.9% S.D.), *Faecalibacterium* (4.5 ± 4.8% S.D.), and *Blautia* (4.3 ± 5.3% S.D.) (Figure S2). For these genera we compared their relative abundances among all amoxicillin treated participants, all azithromycin treated participants, and all non-household controls at each time point sampled (Fig. 1). We found that the azithromycin treated participants had a significantly (Kruskal–Wallis with permutation test; *p* < 0.05) lower relative abundance of *Bifidobacteria* compared to controls after starting antibiotic therapy (after day 0). *Bifidobacteria* was also significantly lower in azithromycin treated participants than the amoxicillin treated participants at day 7 and week 8 (*p* < 0.001). Additionally, we observed that at day 7 and week 8 the relative abundance of *Bacteroides* was significantly higher (*p* < 0.05) in the azithromycin treated participants compared to the non-household controls. The amoxicillin treated participants had a significantly higher (*p* < 0.05) relative abundance of *Bacteroides* compared to controls only on day 3.

**Fig. 2:**
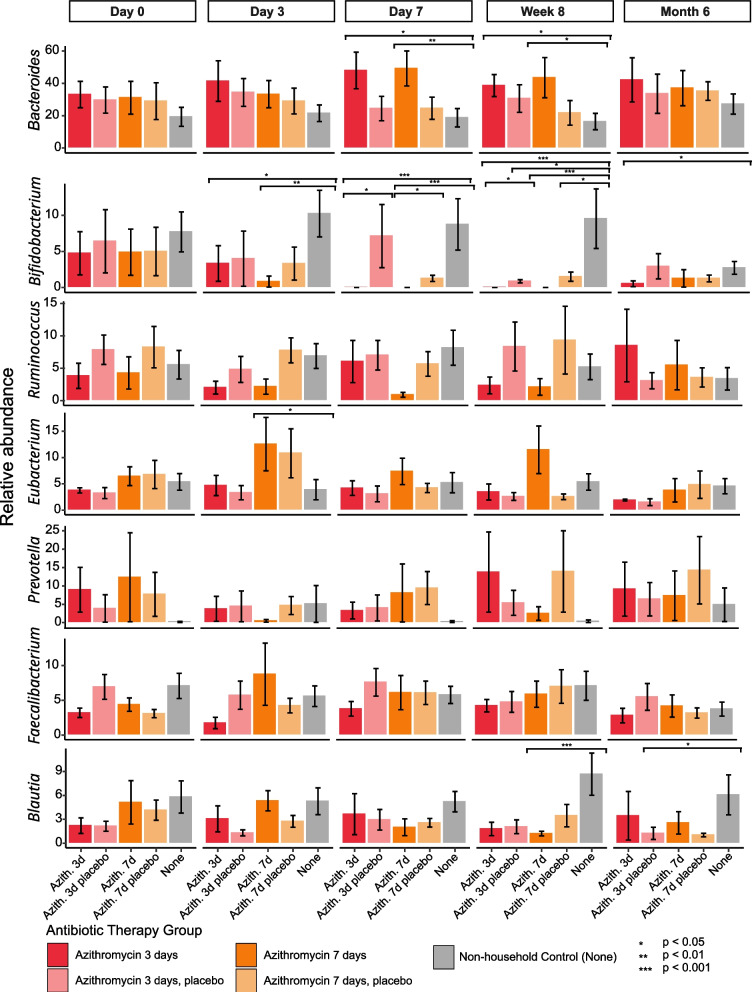
Relative abundance (± standard error) of the most dominant bacterial genera among participants with 7-day azithromycin therapy (Azith 7d) and 3-day azithromycin therapy (Azith 3d) and their household and non-household controls. The y-axis represents the relative abundace of the dominant bacterial genera, and the x-axis represents the therapy they received, grouped by the time point sampled. Bars are colored by the therapy they received (Azith 7d, dark orange; Azith 3d, dark red; Azith 7d household controls, light orange; Azith 3d household controls light red; non-household controls, gray). *denotes significance based on Kruskal–Wallis tests with correction via the Holm method

We then focused on the azithromycin treated participants and compared the 7-day (Azith 7d) and 3-day trials (Azith 3d) with their household and non-household controls (Fig. 2). *Bifidobacteria* was significantly lower at all time points following day 0 in the Azith 3d participants compared to non-household controls. For the Azith 7d participants *Bifidobacteria* was significantly lower in relative abundance compared to the non-household controls at day 3, day 7, and week 8. They were also significantly lower than their housemates at day 7.

Again, we observed that at day 7 and week 8 the relative abundance of *Bacteroides* was significantly higher in both the Azith 3d and Azith 7d treated participants compared to the non-household controls. Interestingly, the housemates of both Azith 3d and Azith 7d treated participants that were given placebo treatment exhibited statistically significant lower relative *Bifidobacterium* (Kruskal–Wallis; *p* < 0.05) abundance at week 8. This difference was also seen in the housemates of the Azith 7d treated patients at day 7 but was above the significance cutoff. The same process was conducted for the amoxicillin treated participants, with the only significance determined to be the Amox 3d participants having a higher relative abundance of *Bacteroides* compared to non-household controls on day 3 (Figure S3).

Using a Spearman’s test we also determined whether there was any correlation between the relative abundance of the seven most dominant genera and duration of antibiotic use (between day 0 and day 7) within each group. These results agreed with our previous analyses with regard to the abundance of *Bifidobacteria.* We found that the abundance of *Bifidobacteria* decreased significantly between days 0 and 7 for both the Azith 3d (Spearman*; R* =  − 0.6, *p* = 0.0081) and the Azith 7d (Spearman; *R* =  − 0.71, *p* = 0.00086) participants. There was no significant change for either the household or non-household controls. Taken together, these data show the impact of azithromycin use of the gut microbiome, predominantly with regard to *Bifidobacteria.*

In addition, to identify specific bacterial species that had a relative abundance significantly associated with antibiotic use at each time sampled, we used multivariate association with linear models (MaAsLin2) pipeline controlling for age, sex, and race and corrected for multiple comparisons. This pipeline was run on all azithromycin treated participants, all azithromycin household controls and all non-household controls. Using this method, the only significant associations were detected between azithromycin treated participants and non-household controls at day 7 for two bacterial species. *Bacteroides vulgatus* was significantly (*p* = 0.022, coefficient -0.063) higher and *Bifidobacterium longum* was significantly (*p* = 0.004, coefficient 0.025) lower in the azithromycin treated participants (Figure S4). This pipeline was also run on all amoxicillin treated participants, all amoxicillin household controls, and all non-household controls with no significant changes in relative abundances identified.

### Shifts in Antibiotic Resistance Genes (ARGs)

**Fig. 3:**
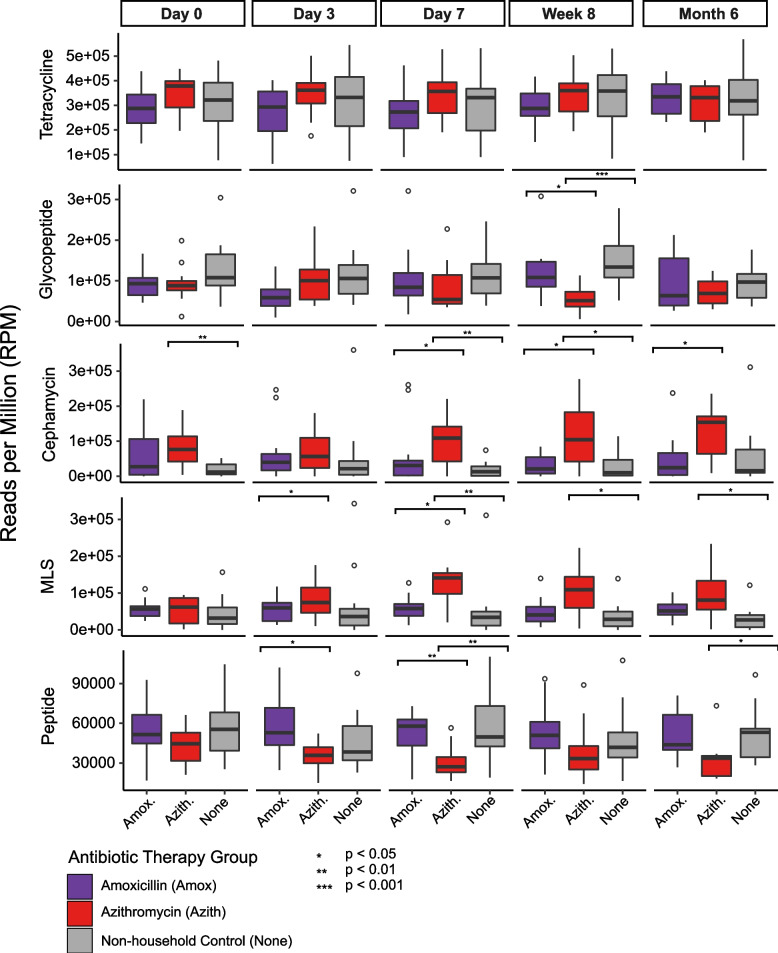
Relative abundance of the most dominant ARG drug classes among all amoxicillin treated participants, all azithromycin treated participants, and all non-household controls at each time point sampled. The y-axis represents the relative abundace of the dominant drug classes, and the x-axis represents the therapy they received grouped by the time point sampled. Boxplots are colored by their treatment status (amoxicillin, purple; azithromycin, red; non-household controls, gray). Abundace calcuated via reads per million (RPM) metric. *denotes significance based on Kruskal–Wallis tests with correction via the Holm method

To assess antibiotic resistance in the samples before, during, and after antibiotic therapy we conducted a BLASTX analysis of reads against the CARD database [24]. In total we identified over 314 ARGs, conferring resistance to over 81 drug classes/combination of drug classes, as designated by CARD. To determine a normalized abundance for each ARG we utilized the reads per million (RPM) metric. The drug classes that were on average 2% or greater included: tetracycline (31.6 ± 10.4% S.D.), glycopeptide (10.6 ± 6.1% S.D.), cephamycin (6.9 ± 7.8% S.D.), MLS (macrolide, lincosamide, streptogramin) (5.6 ± 5.8% S.D.), peptide (5 ± 2.3% S.D.), rifamycin (4.8 ± 2.3% S.D.), aminoglycoside (4.7 ± 4.8% S.D.), macrolide (3.7 ± 3.4% S.D.), diaminopyrimidine (2.4 ± 3.1% S.D.), cephalosporin (2.4 ± 2.6% S.D.), lincosamide (2.3 ± 2.5% S.D.), and glycylcycline; tetracycline (2.1 ± 3.3% S.D.) (Figure S5). MLS resistance was primarily represented by changes in the relative abundance of the *erm* gene. Similar to what we conducted for the bacterial relative abundances, we compared the ARG drug classes whose abundance was > 5% among all amoxicillin treated participants, all azithromycin treated participants, and all non-household controls at each time point sampled (Fig. 3). We observed that the azithromycin treated participants had a significantly higher abundance of MLS ARGs compared to non-household controls at days 7, week 8, and month 6 (Kruskal–Wallis; *p* < 0.05). Conversely, the azithromycin treated participants had a lower abundance of glycopeptide ARGs at week 8 and peptide ARGs at day 7 and month 6.

**Fig. 4:**
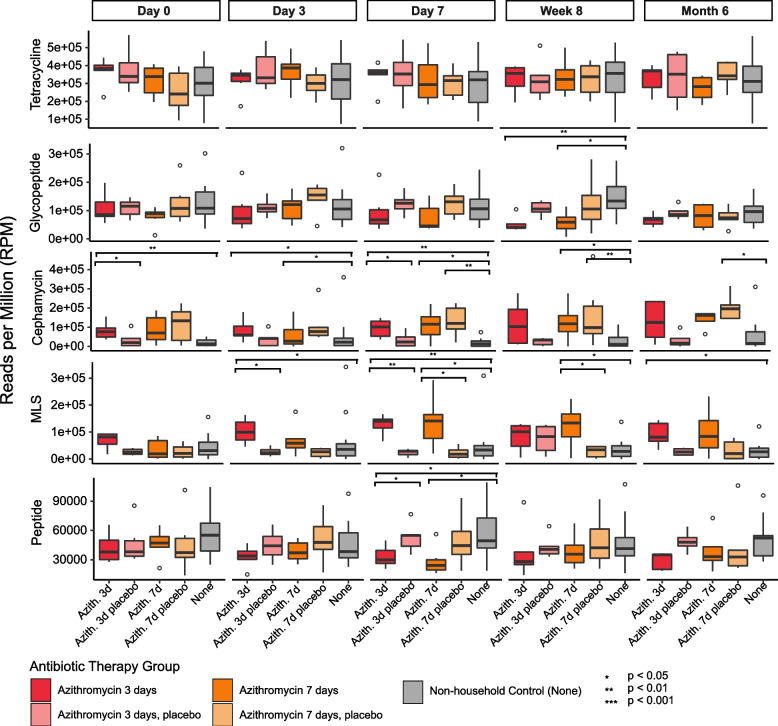
Relative abundance of the most dominant ARG drug classes among participants with 7-day azithromycin therapy (Azith 7d) and 3-day amoxicillin therapy (Azith 3d) and their household and non-household controls at each time point sampled. The y-axis represents the relative abundace of the dominant drug classes, and the x-axis represents the therapy they received grouped by the time point sampled. Boxplots are colored by their treatment status (Azith 7d, dark orange; Azith 3d, dark red; Azith 7d household controls, light orange; Azith 3d household controls light red; non-household controls, gray). Abundance calculated via reads per million (RPM) metric. *denotes significance based on Kruskal–Wallis tests with correction via the Holm method

When parsing the antibiotic treatment groups by length of therapy and adding the household controls, we observed a similar pattern (Fig. 4). For the Azith 7d participants MLS ARGs were significantly higher in abundance compared to both the household and the non-household controls at day 7 and week 8. For the Azith 3d participants the MLS ARGs were significantly higher in abundance compared to both the household and the non-household controls at days 3 and 7.

Again, using a Spearman’s test we determined whether there was any correlation between the abundance of ARG drug classes and duration of antibiotic use (between day 0 and day 7) within each group. We found that the abundance of the MLS ARGs increased significantly between days 0 and 7 for both the Azith 3d (Spearman*; R* = 0.58, *p* = 0.012) and the Azith 7d (Spearman*; R* = 0.51, *p* = 0.03) participants. The only other significant drug class was the peptide ARGs, which decreased significantly in the Azith 7d (Spearman*; R* =  − 0.47, *p* = 0.048) participants. There was no significant change for either the household or non-household controls.

### Changes in phage community composition in response to antibiotics

**Fig. 5:**
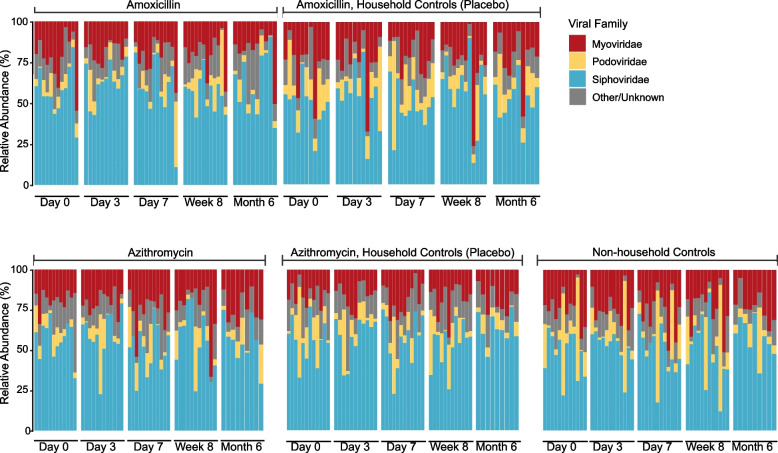
Relative abundace of the dominant bacteriophage families over time and antibiotic treatment. The y-axis represents the realtive abundace of the dominant phage families, and the x-axis represents the different subjects grouped by time and the therapy they received. Groups that received antibiotics, placebo (household controls), or no therapy (controls) are labeled accordingly

In order to more faithfully mine virus taxa from the metagenomic data, reads were assembled into contigs. Not surprisingly, most of the phage contigs were from the three major families of Caudovirales (Fig. 5). Using a Spearman’s test we determined for all groups that there were no significant correlations between the abundance of the three major families of Caudovirales and duration of antibiotic use (between day 0 and day 7).

## Discussion

The purpose of this study was to evaluate the effects of two of the most commonly used antibiotics in the U.S., amoxicillin and azithromycin, on the microbiome of inhabitants in the same household. While prior studies, including our own, have demonstrated that these antibiotics can impact the microbiome [20], in this study, we use metagenomics to gain genome-level insights into the microbiome and patterns of antimicrobial resistance. In our prior study using this same cohort of individuals, we used 16S rRNA to evaluate changes in the microbiome of individuals and their household members but did not have genome-level insights into particular organisms that were impacted by the use of the antibiotics [20]. In that study, we determined that there were significant and long-lasting impacts not only on the fecal microbiome, but also on the salivary microbiome in response to antibiotics, but the greatest impacts were observed on the fecal microbiome. For that reason, we focused the efforts of this study on the fecal microbiome to identify which individual microbes and antibiotic resistance gene markers may be impacted by these antibiotic courses.

In clinical medicine, we recognize the impacts of amoxicillin on our commensal microbes. While previously considered a broad-spectrum antibiotic, the abundance of beta lactamases present in human pathogens and commensals makes its impact more difficult to predict [25, 26]. The antibiotic has impacts on gram positive, gram negative, and anaerobic microorganisms that do not possess beta lactamases. It also has a relatively short half-life compared to azithromycin, which has an extended half-life, which could result in more long-lasting effects on the microbiome [27]. While these antibiotics do not have the same spectrums of activity, there is some overlap in the microorganisms they target [28]. Our major findings were different for each antibiotic, with there being a significant and sustained reduction in *Bifidobacterium* species in response to the azithromycin therapy. We also identified an increase in the relative abundance of *Bacteroides* species. While the increase in *Bacteroides* may be a specific response to the reduction of *Bifidobacteria,* more studies are needed to determine possible causation. At Week 8 post-treatment with both amoxicillin and azithromycin we noticed a significant decrease in *Blautia* species, which remained slightly decreased at 6 months post-treatment. *Blautia* has been found to have health benefits [26], including a negative association with obesity [29, 30] and inflammatory disease [31]. That some of the most abundant bacteria in the gut are impacted in response to these antibiotics, suggests that the effects of these antibiotics on gut microbiome health may be substantial and long-lasting.

We also saw a significant decrease in the relative abundance of *Bifidobacterium* in the placebo-treated housemates of the Azith 3d and Azith 7d treated participants. *Bifidobacterium* are among the taxa previously identified as often being shared between spousal pairs and associated with reduced disease incidence and severity [9], but the previous study did not include the analysis of the effect of microbiome perturbations on the microbial communities. Here, we provide evidence of azithromycin treatment resulting in the reduction in *Bifidobacterium* in not only the treated individual, but also their close housemate months after the conclusion of the antibiotic regimen. Given the association of stable *Bifidobacteria* populations with improved gut health [32] Fthese results could have important implications of the gut health of more than just the individual receiving antibiotic treatment. No other significant changes were observed in members of the microbial community, which highlights how stable the microbiomes were. The reduction of *Bifidobacteria* in the non-treated roommate requires further investigation. One possibility is the transmission of *Bifidobacteria* lytic phages from the treated roommate, however this was not supported in the virome analysis. An alternative explanation is noise in the system since *Bifidobacteria* relative abundances were low.

The antibiotic resistance class that was enriched in the azithromycin treated patients was the MLS class that includes macrolides, lincosamides, and streptogramins. Although they have different chemical structures, MLS antibiotics have a similar mode of action. They inhibit protein synthesis by binding to overlapping sites on the 50S ribosomal subunit. The predominant mechanisms of resistance to MLS antibiotics are target modification through methylation of rRNA, active efflux and antibiotic inactivation [33]. In this case, we found that *erm* genes were specifically enriched. These genes function by dimethylation of a single adenine in the 50S ribosomal subunit, leading to cross-resistance of the 3 drugs classes. While the *erm* gene is probably best known for its role in erythromycin resistance, it has also been shown to confer resistance to azithromycin [34]. We found that the MLS drug classes were significantly and sustainably higher compared to both the household and non-household controls, suggesting its change in abundance was a direct response to azithromycin therapy.

Because we previously identified differences amongst the viral communities within a household [17], we also characterized some elements of the virome communities in this study. In our prior study, we characterized the relative proportions of the virome that were shared within a household and found that viruses likely were commonly shared amongst household members. We did not identify trends in that study that were associated with antibiotic use. In this study, we characterized the viral community in a different manner by characterizing those viruses we could identify from metagenome reads and assembling them into larger viral contigs. We found bacteriophages in the fecal community mostly from the *Caudovirales* families *Siphoviridae*, *Myoviridae*, and *Podoviridae* (Fig. 5). Similarly to our previous study, we did not observe any significant correlations between these viral families and duration of antibiotic use. However, specifically targeting the viral population through chemical or mechanical concentration may provide a more accurate picture of the potential changes brought on by antibiotic use.

In our analyses of the longitudinal data, we only examined relationships across the different groups within each assessment time. Although we did find differences of interest for our hypothesized relationships in response to azithromycin (macrolide antibiotic), we were not able to model and test if such differences changed over time. In future studies, we will employ longitudinal models such as the generalized estimating equations to examine such temporal trends.

## Conclusions

As we characterize the microbiome of the gut, there is still much we do not know about the responses to antibiotic perturbations. Prior studies have demonstrated that there can be long-term impacts of commonly used antibiotics on the gut microbiome [35, 36], and also have suggested that specific microbes in the gut may be shared between individuals in close contact [17]. While much has been revealed over time about the impacts of common antibiotics on the gut microbiome, there still is a knowledge gap as to the potential medium-term changes that can be observed in individuals taking the same antibiotic. We performed this study using metagenomics of the gut microbiome to further our understanding of specific microbes that may be impacted by common antibiotics. We found that *Bifidobacterium* was significantly impacted in individuals taking the antibiotic azithromycin. These observations were not found in non-household controls, which provides some assurance that the effects of antibiotics can be individual-specific.

## Methods

### Cohort design

This study was retrospectively registered as a clinical trial (NCT05169255). It was not designed to conform to CONSORT guidelines; however, does conform to many of them. Forty-eight subjects were enrolled in the study in pairs, with 2 individuals living in each household. An additional 8 individuals were enrolled without a housemate and received no therapy over the course of the 6-month study. Households were randomized into either the amoxicillin or azithromycin arms of the study. Those subjects also were randomized to receive either antibiotic or placebo; however, because of the large numbers of penicillin allergies reported (Table S1) and subjects using oral contraceptives (interact with azithromycin), some subjects who were randomized to receive antibiotics were given the placebo, while their housemate received the antibiotic instead. Of the household pairs, 6 pairs were placed into the 3-day amoxicillin arm, 6 pairs were placed into the 7-day amoxicillin arm, 6 pairs were placed into the 3-day azithromycin arm, and 6 pairs were placed into the 7-day azithromycin arm (Fig. 1). In each household, 1 subject received either 3 or 7 days of an antibiotic and the other subject received either 3 or 7 days of the placebo (vitamin C). Vitamin C was used as placebo since it is conveniently packaged and it has no safety concerns. The dose of amoxicillin was 500 mg twice daily, and the dose of vitamin C was 500 mg twice daily. The dose of azithromycin was 500 mg on the first day, and 250 mg daily thereafter (this dosing was used to be consistent with the commonly prescribed Z-Pak). In the azithromycin arm, the placebo was given at 500 mg once daily. Each subject enrolled donated feces on day 0 (day prior to antibiotics), day 3 (3 days after initiation of antibiotics), day 7, week 8, and month 6. Of the 24 households enrolled, 5 of those households were lost to follow-up and did not provide specimens at the month 6 time point. Each subject provided fecal specimens that were immediately frozen at -20 °C prior to transporting on ice to the study site where they were frozen ad -80 °C until use in this study. They were encouraged to provide specimens in the AM prior to breakfast to facilitate their use in this study. Exclusion criteria included prior antibiotic use for 1 year prior to the initiation of the study, and preexisting medical conditions such as diabetes, inflammatory bowel disease, and organ transplantation that might result in significant immunosuppression. All subjects self-reported their health status and were genetically unrelated.

#### Sequence processing

After sequencing the paired-end reads were quality trimmed using Trimmomatic ver. 0.39 (sliding window:4:30 min len:60) [37] and then merged with FLASH ver. 1.2.11 [38]. Reads were then mapped to the human genome with inclusive parameters via Bowtie2 ver. 2.3.5.1 and Samtools ver 1.7, and any subsequent human reads were removed [39, 40].

#### Taxonomic and ARG assignments

Filtered and quality reads were taxonomically profiled using MetaPhlAn2 with default parameters [41]. The genera with the highest relative abundances across samples were used for detailed analysis, a cutoff of an average relative abundance of 4% was used. In addition, filtered and quality reads searched against the “Comprehensive Antibiotic Resistance Database” (CARD; retrieved May 2020) via DIAMOND BLASTX (ver. 0.9.24.125) (E value ≤ 1e-5) [24, 42]. Reads were considered an ARG if it had > 40% coverage and > 80% amino-acid identity to a CARD protein [43, 44]. A normalized abundance was calculated for all ARGs with a minimum of 10 assigned reads via the reads per million (RPM) metric, which considers both gene length and sampling depth [40, 45].

#### VirSorter

Quality reads were then assembled de novo with MEGAHIT [46]. Viral contigs were mined from each assembled library using VirSorter. Contigs classified as category 1 (“most confident” predictions) and category 2 (“likely” predictions) were then subjected to a protein- BLAST (tBLASTx. 2.6.0 +) (E value ≤ 1e-5) against the NCBI viral database. Abundance was calculated for each contig by recruiting quality-controlled reads to assembled contigs using Bowtie2 ver. 2.3.5.1 and then using the “depth” function of Samtools ver 1.7 to compute the per-contig coverage [39]. To normalize abundances across libraries, contig coverages were divided by the sum of coverage per million, similar to the TPM metric used in RNA-Seq [40, 45]. Scripts performing these assignments and normalization are available at https://github.com/dnasko/baby_virome.

#### Statistics

Comparisons of the relative abundances of the seven dominant bacterial genera and the abundance of the dominant ARG drug classes were assessed using Kruskal–Wallis with inference based on permutation tests and multiple-comparison [46] correction via the Holm method [47]. To identify specific bacterial species that had a relative abundance significantly associated with antibiotic use at each time sampled, we used multivariate association with the linear mixed-effect model (MaAsLin2) controlled for multiple comparison via FDR [48]; Fixed effects: antibiotic treatment, age, race, and sex; random effects: subject [49]. Permutation-based inference was used to improve inference validity. To test for differences among the subject demographics by treatment group we used an ANOVA and Fisher exact test of independence for numerical and categorical data, respectively. All tests were run in RStudio Version 1.0.153.

## Supplementary Information

**Additional file 1:** **Table S1.** Study Subjects.

**Additional file 2:** **FigureS1.** Study design. Schematic of subjects enrolled in the study. Figure adapted from Abeles et al, 2016.

**Additional file 3:** **FigureS2.** Relative abundace of the dominant bacterial genera over time and antibiotic treatment. The y-axis represents the realtive abundaceof the dominant bacterial genera (genera >2% on average), and the x-axis represents the different subjects grouped by time and the therapy they received. Groups that received antibiotics, placebo (household controls), or no therapy(controls) are labeled accordingly.

**Additional file 4:** **Figure S3.** Relative abundance (±standarderror) of the most dominant bacterial genera among participants with 7-dayamoxicillin therapy (Amox 7d) and 3-day amoxicillin therapy (Amox 3d) and theirhousehold and non-household controls. The y-axis represents the relativeabundance of the dominant bacterial genera, and the x-axis represents thetherapy they received, grouped by the time point sampled. Bars are colored bythe therapy they received (Amox 7d, dark blue; Amox 3d, dark purple; Amox 7dhousehold controls, light blue; Amox 3d household controls light purple;non-household controls, gray). *denotes significance based on Kruskal-Wallistests with correction via the Holm method.

**Additional file 5:** **Figure S4. **Boxplot of the significantly different bacterial species as reported by the MaAsLin2 pipeline controlling for age, sex, and race. Boxplots are colored by their treatment status (azithromycin, red; household control, light red; non-household control, gray).

**Additional file 6:** **Figure S5.** Abundance (±standard error) of the most dominant ARG drug classes over time for all antibiotic treatment groups. The y-axis represents the relative abundance of the dominant drug classes, and the x-axis represents the time point sampled. Line graphs are grouped by their treatment status. Abundance calculated via reads per million (RPM) metric.

**Additional file 7:** **FigureS6.**Antibiotic mechanisms abundances.  Abundance(±standard error) of the most dominant ARG drug classes. Abundance calculatedvia reads per million (RPM) metric.

## Data Availability

The datasets used and/or analyzed during the current study are available in the Sequence Read Archive under Bioproject ID# PRJNA715245 available at https://www.ncbi.nlm.nih.gov/bioproject/PRJNA715245

## Abbreviations

CDC: Centers for disease control
ARGs: Antibiotic resistance genes
CARD: Comprehensive antibiotic resistance database
RPM: Reads per million
Azith: Azithromycin
Amox: Amoxicillin
