# Supplementary material for: Common antibiotics, azithromycin and amoxicillin, affect gut metagenomics within a household
Source: BMC Microbiol. 2023 Aug 2;23:206. doi: 10.1186/s12866-023-02949-z (PMC10394940; doi:10.1186/s12866-023-02949-z)
Supplement: Supplementary file 1 — Additional file 1: Table S1. Study Subjects. [file 12866_2023_2949_MOESM1_ESM.pdf]

Table S1: Study Subjects

Subjects	Antibiotic	Days of therapy	Medication Allergies
CA05	Amoxicillin	7	None
CA06	Placebo	7	Penicillin
CA07	Amoxicillin	7	None
CA08	Placebo	7	Penicillin
CA09	Amoxicillin	3	None
CA10	Placebo	3	None
CA11	Azithromycin	7	None
CA12	Placebo	7	None
CA13	Amoxicillin	3	None
CA14	Placebo	3	None
CA15	Azithromycin	7	None
CA16	Placebo	7	None
CA17	Amoxicillin	3	None
CA18	Placebo	3	None
CA19	Amoxicillin	3	Sulfa
CA20	Placebo	3	Penicillin
CA21	Amoxicillin	7	None
CA22	Placebo	7	None
CA23	Amoxicillin	7	None
CA24	Placebo	7	Penicillin
CA25	Amoxicillin	3	None
CA26	Placebo	3	None
CA27	Amoxicillin	7	None
CA28	Placebo	7	Penicillin
CA29	Amoxicillin	7	None
CA30	Placebo	7	None
CA31	Amoxicillin	3	None
CA32	Placebo	3	None
CA33	Azithromycin	3	None
CA34	Placebo	3	Penicillin
CA35	Azithromycin	3	None
CA36	Placebo	3	None
CA37	Azithromycin	7	None
CA38	Placebo	7	None
CA39	Azithromycin	7	None
CA40	Placebo	7	None
CA43	Azithromycin	3	None
CA44	Placebo	3	None
CA47	Azithromycin	7	None
CA48	Placebo	7	None
CA49	Azithromycin	3	None
CA50	Placebo	3	None
CA53	Azithromycin	7	None
CA54	Placebo	7	None

CA55	Azithromycin	3	None
CA56	Placebo	3	None
CA57	Azithromycin	3	None
CA58	Placebo	3	None
CA02	None	0	None
CA81	None	0	None
CA83	None	0	None
CA84	None	0	None
CA85	None	0	None
CA86	None	0	None
CA87	None	0	None
CA89	None	0	None
