# Supplementary figures and images for: Common antibiotics, azithromycin and amoxicillin, affect gut metagenomics within a household
Source: BMC Microbiol. 2023 Aug 2;23:206. doi: 10.1186/s12866-023-02949-z (PMC10394940; doi:10.1186/s12866-023-02949-z)

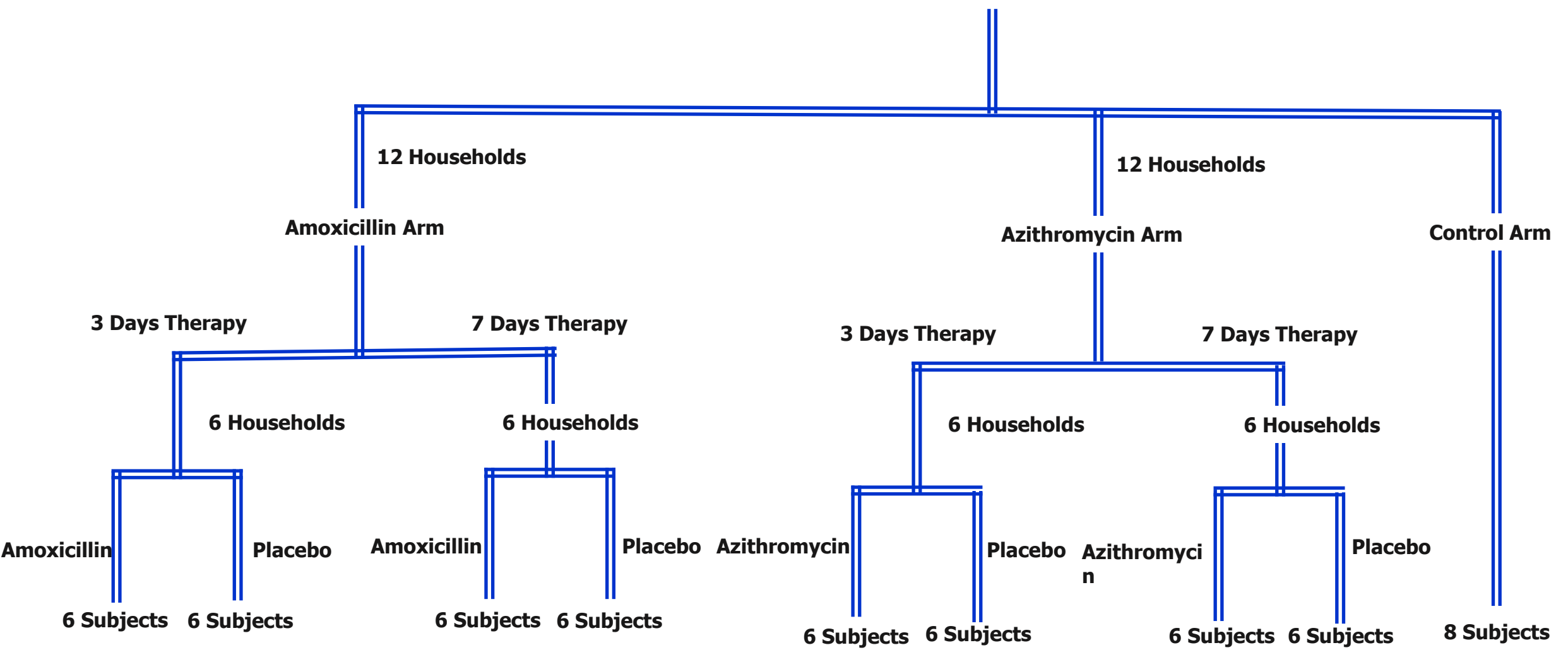

Supplement: Supplementary file 2 — Additional file 2: FigureS1. Study design. Schematic of subjects enrolled in the study. Figure adapted from Abeles et al, 2016. [file 12866_2023_2949_MOESM2_ESM.pdf]

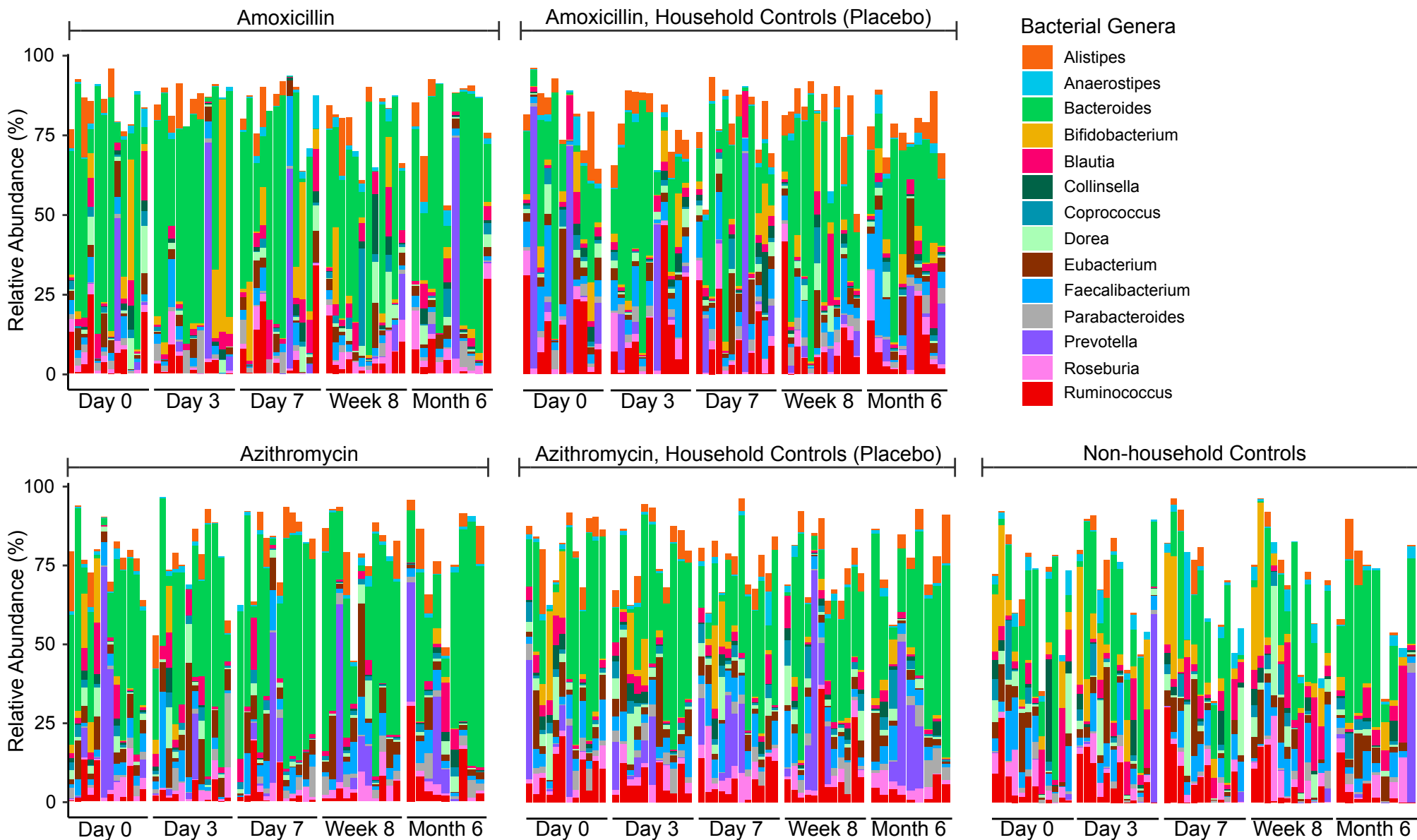

Supplement: Supplementary file 3 — Additional file 3: FigureS2. Relative abundace of the dominant bacterial genera over time and antibiotic treatment. The y-axis represents the realtive abundaceof the dominant bacterial genera (genera >2% on average), and the x-axis represents the different subjects grouped by time and the therapy they received. Groups that received antibiotics, placebo (household controls), or no therapy(controls) are labeled accordingly. [file 12866_2023_2949_MOESM3_ESM.pdf]

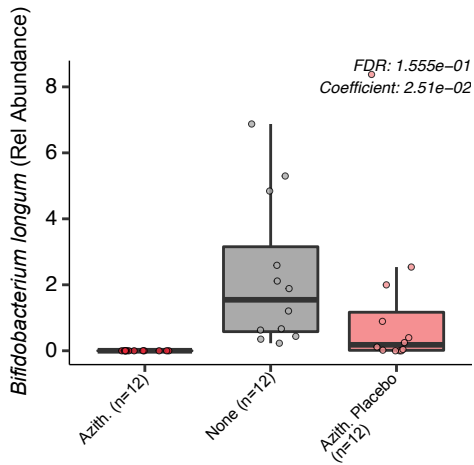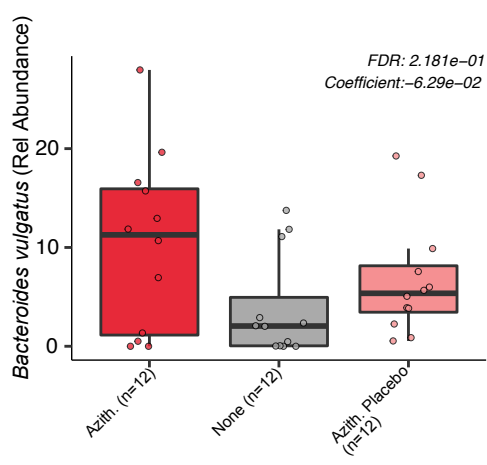

Supplement: Supplementary file 4 — Additional file 4: Figure S3. Relative abundance (±standarderror) of the most dominant bacterial genera among participants with 7-dayamoxicillin therapy (Amox 7d) and 3-day amoxicillin therapy (Amox 3d) and theirhousehold and non-household controls. The y-axis represents the relativeabundance of the dominant bacterial genera, and the x-axis represents thetherapy they received, grouped by the time point sampled. Bars are colored bythe therapy they received (Amox 7d, dark blue; Amox 3d, dark purple; Amox 7dhousehold controls, light blue; Amox 3d household controls light purple;non-household controls, gray). *denotes significance based on Kruskal-Wallistests with correction via the Holm method. [file 12866_2023_2949_MOESM4_ESM.pdf]

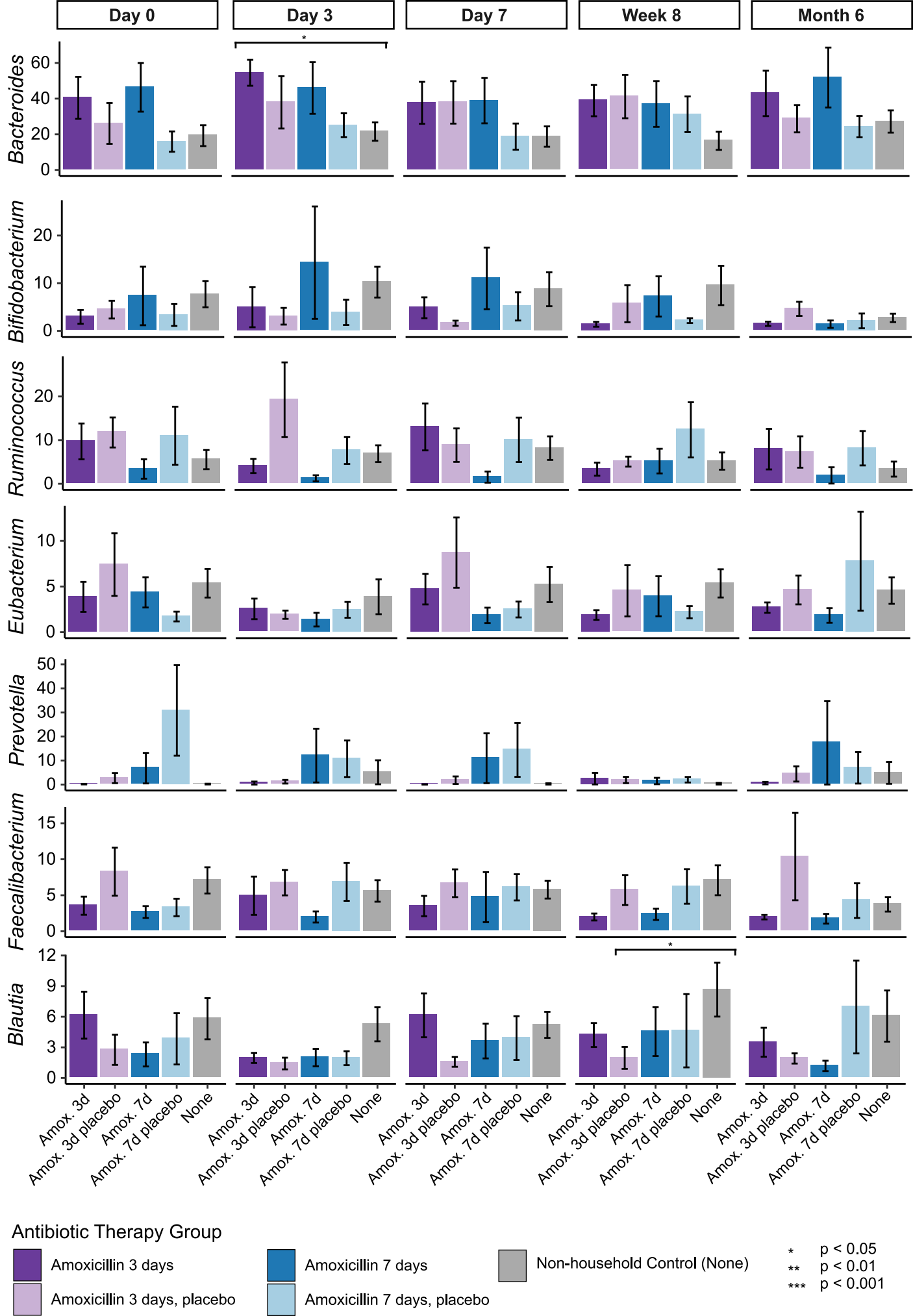

Supplement: Supplementary file 5 — Additional file 5: Figure S4. Boxplot of the significantly different bacterial species as reported by the MaAsLin2 pipeline controlling for age, sex, and race. Boxplots are colored by their treatment status (azithromycin, red; household control, light red; non-household control, gray). [file 12866_2023_2949_MOESM5_ESM.pdf]

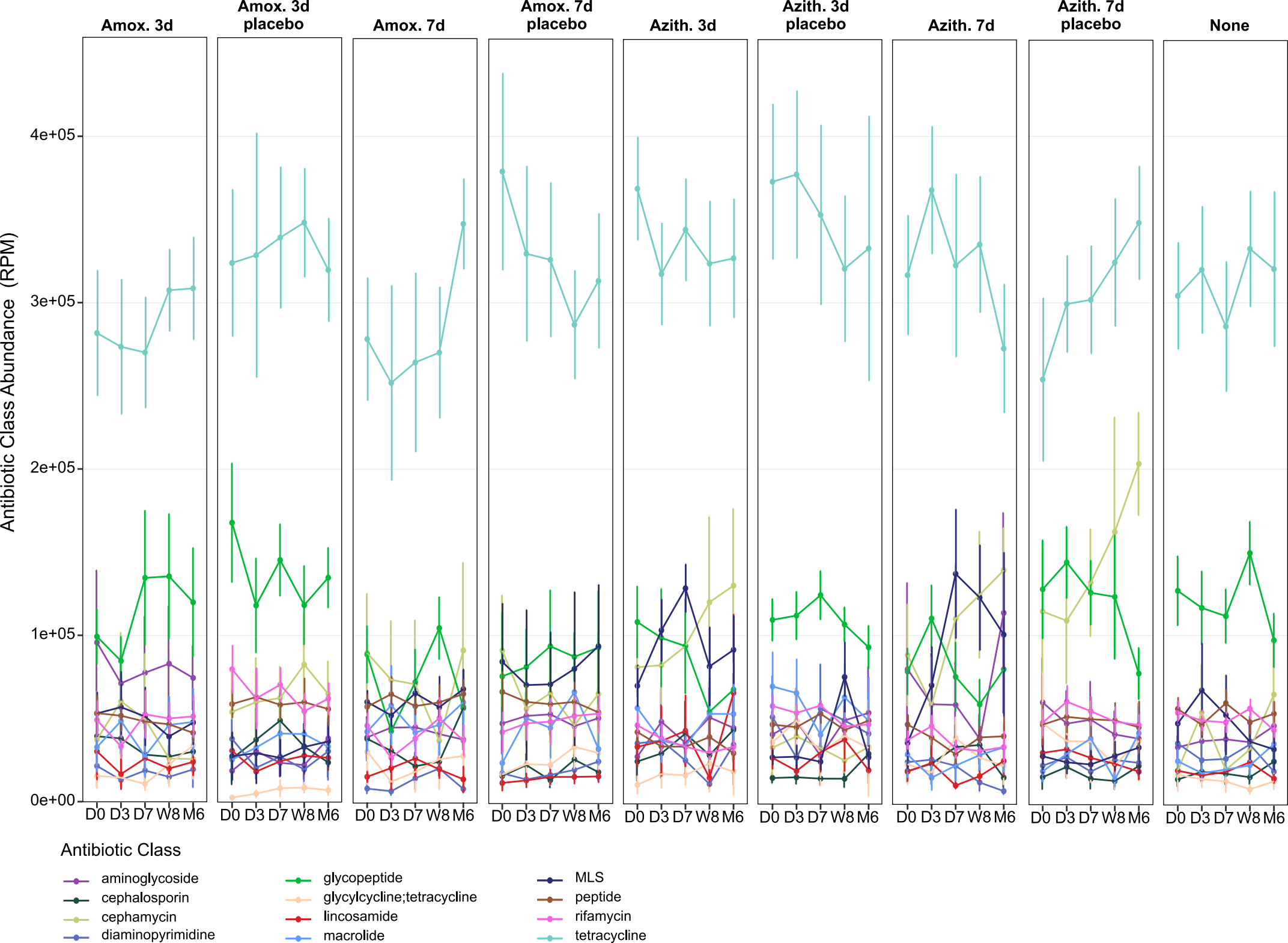

Supplement: Supplementary file 6 — Additional file 6: Figure S5. Abundance (±standard error) of the most dominant ARG drug classes over time for all antibiotic treatment groups. The y-axis represents the relative abundance of the dominant drug classes, and the x-axis represents the time point sampled. Line graphs are grouped by their treatment status. Abundance calculated via reads per million (RPM) metric. [file 12866_2023_2949_MOESM6_ESM.pdf]

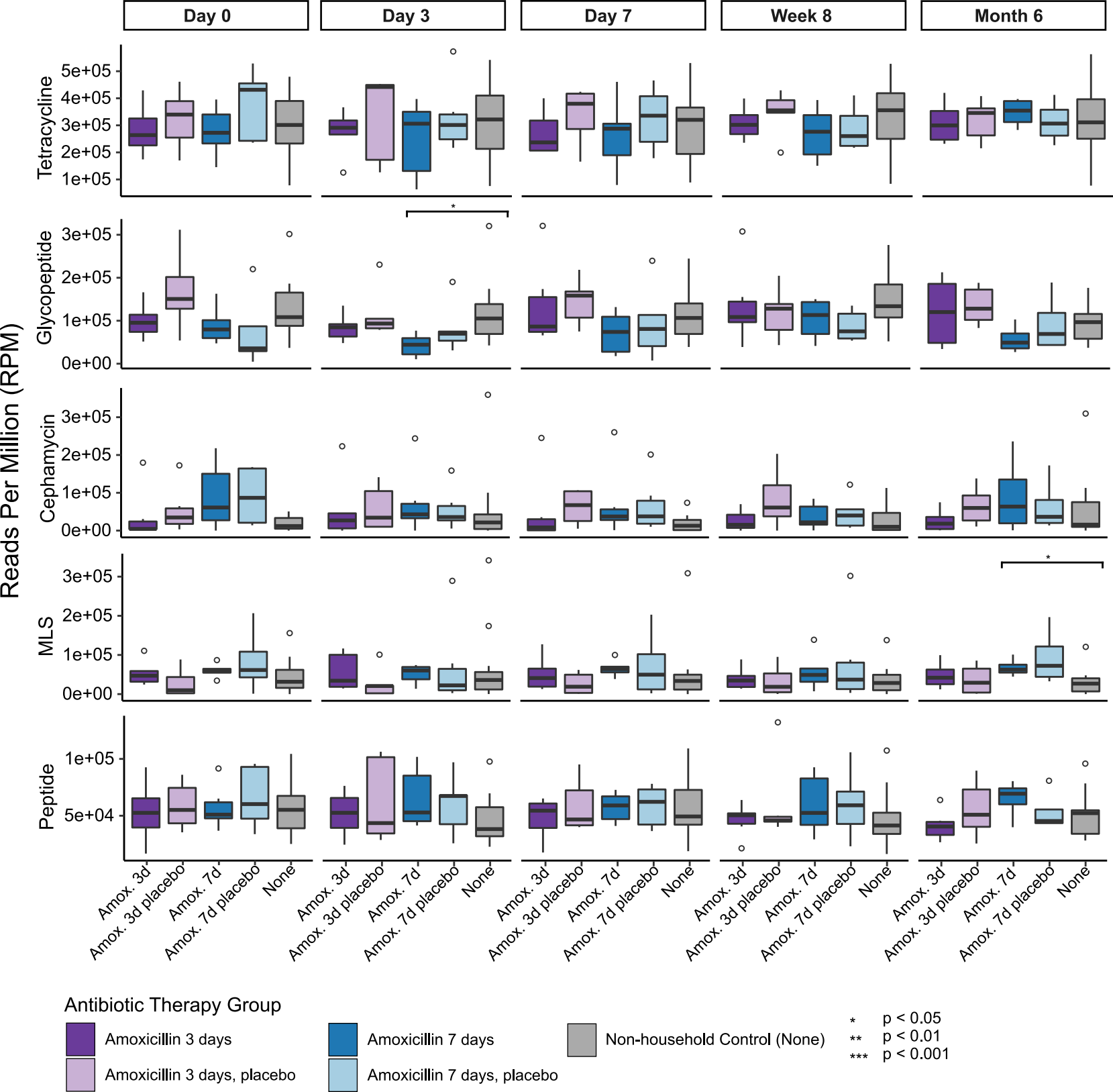

Supplement: Supplementary file 7 — Additional file 7: FigureS6.Antibiotic mechanisms abundances. Abundance(±standard error) of the most dominant ARG drug classes. Abundance calculatedvia reads per million (RPM) metric. [file 12866_2023_2949_MOESM7_ESM.pdf]
